# Urticarial Vasculitis-Associated Intestinal Ischemia

**DOI:** 10.1155/2016/8603679

**Published:** 2016-04-17

**Authors:** Uni Wong, Harris Yfantis, Guofeng Xie

**Affiliations:** ^1^Division of Gastroenterology and Hepatology, University of Maryland Medical Center, Baltimore, MD 21201, USA; ^2^Department of Pathology, Veterans Affairs Maryland Health Care System, University of Maryland School of Medicine, Baltimore, MD 21201, USA; ^3^Division of Gastroenterology and Hepatology, Veterans Affairs Maryland Health Care System, University of Maryland School of Medicine, Baltimore, MD 21201, USA

## Abstract

Urticarial vasculitis (UV) is a rare small vessel vasculitis. UV is often idiopathic but can also present in the context of autoimmune disorders such as systemic lupus erythematosus, drug reactions, infections, or a paraneoplastic syndrome. Extracutaneous complications include intestinal ischemic injuries, in UV patients with nonspecific gastrointestinal symptoms such as abdominal pain and nausea. Prompt recognition and treatment can minimize morbidity and mortality. This paper describes a case of urticarial vasculitis-associated intestinal ischemia.

## 1. Introduction

Urticarial vasculitis (UV) is a rare diagnosis with incidence rate of 4.5 per 100,000 person-years [1]. Majority of cases reported are idiopathic, but UV has been associated with autoimmune disorders, infections, drug reactions, and paraneoplastic syndrome [2]. The condition is characterized by cutaneous manifestations of urticaria along with histopathologic findings of leukocytoclastic vasculitis of the small vessels [3–5]. Pathogenesis of UV is thought to be due to formation and attachment of immune complexes in the walls of small blood vessels, resulting in angioedema, ischemia, and/or necrosis. As a result, clinical manifestations of UV can vary, ranging from urticaria with minimal extracutaneous involvement to a more severe form where patients can have minimal urticaria but have life-threatening multiorgan dysfunction [2]. It can be further classified into normocomplementemic and hypocomplementemic where those patients with low complement levels tend to have poorer prognosis [2]. Therefore, prompt recognition and management of UV are vital in minimizing morbidity and potentially life-threatening complications. We present a case where a patient presenting with abdominal pain was found to have intestinal ischemia in setting of urticarial vasculitis.

## 2. Case Presentation

**Figure 1:**
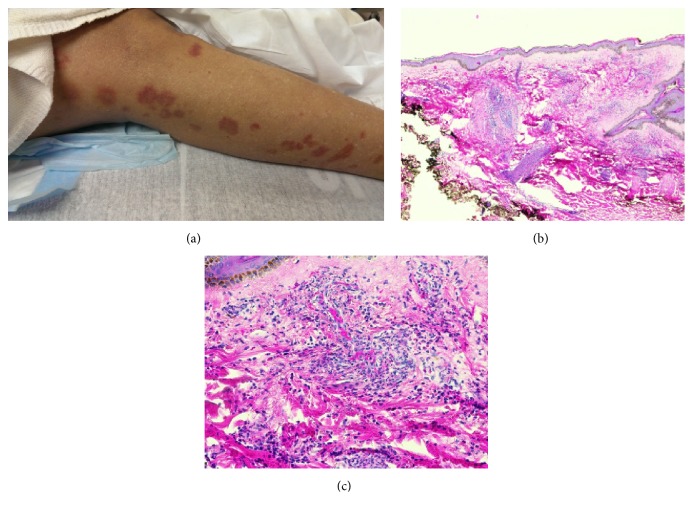
Drug-induced small vessel vasculitis. (a) Skin rash in lower extremities. (b) H&E staining from skin punch biopsy (20x) showing dense infiltrate of neutrophils and eosinophils surrounding vessels with extravasation of red blood cells. (c) H&E staining from the same skin punch biopsy (200x).

A 62-year-old African American woman was seen by the gastroenterology consult service for evaluation of diffuse abdominal pain and nausea. Patient was initially seen in the hospital for an infected wound at her left ankle where she recently had a lipoma resected. After getting an incision and drainage of the infected wound, she was treated with clindamycin that was complicated by a skin rash that developed three days later on her bilateral upper and lower extremities. The rash was characterized as erythematous tender papules that were extremely pruritic (Figure 1(a)). She received hydroxyzine and diphenhydramine for symptomatic management. Her antimicrobial therapy was then switched from clindamycin to doxycycline. Patient subsequently developed persistent diffuse abdominal pain, mild hematochezia, and nausea/vomiting. She denied any fever/chills, night sweats, or weight loss. Pertinent review of systems was otherwise unremarkable.

**Figure 2:**
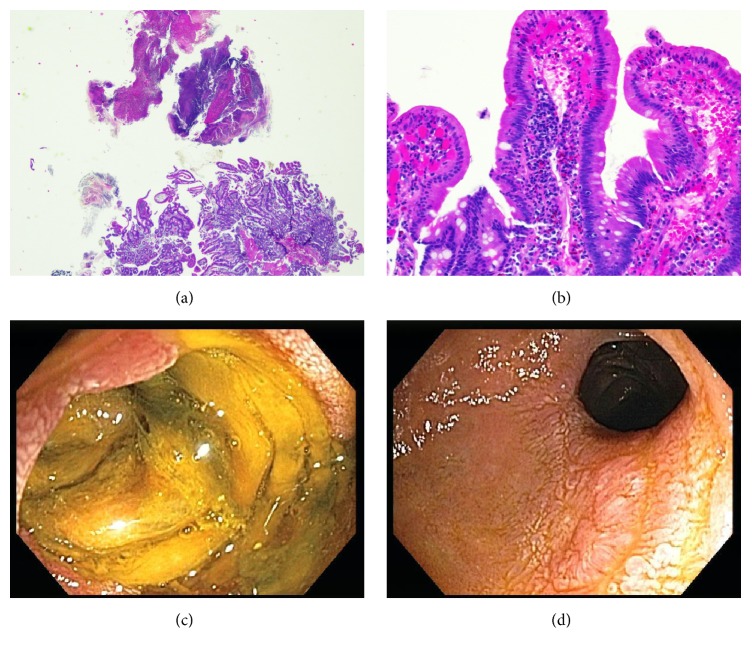
Vasculitis-associated duodenitis. (a) H&E staining from duodenal biopsy (40x) showing fibrinopurulent exudate consistent with ulceration/erosion. (b) H&E staining from duodenal biopsy (200x) showing active duodenitis including neutrophils within the surface epithelium and in the lamina propria surrounding small vessels with capillary ectasia and red blood cell extravasation. (c) EGD image of active duodenitis. (d) EGD image of mild to moderate duodenal stricture.

Workup consisted of an abdominal CT scan which revealed thickening and stranding associated with the distal duodenum and proximal jejunum. There was no evidence of large vessel narrowing or significant atherosclerosis on imaging. Skin biopsy showed small vessel vasculitis (vascular damage with intense infiltrates of neutrophils and scattered eosinophils), consistent with urticarial vasculitis (Figures 1(b) and 1(c)). Serologies obtained to evaluate for concurrent autoimmune disorder, hematological malignancy, or infections including ANA, anti-PR3, anti-TTG, anti-SMA, anti-centromere, anti-gliadin, anti-SSA/SSB, anti-RNP, anti-MPO, anti-dsDNA, c-ANCA and p-ANCA, SPEP/UPEP, HIV, viral hepatitis panel, serum IgG, and cryoglobulin levels were all negative or normal. Complement levels (C3 and C4) were normal. Other infectious workups including C. Diff. and TB were also negative. Esophagogastroduodenoscopy (EGD) performed a week after onset of her GI symptoms revealed severe circumferential ulcerations at the third portion of duodenum (D3) (Figures 2(c) and 2(d)). Histology on endoscopic biopsy showed acute duodenitis with focal necrosis with eosinophils, consistent with intestinal ischemia caused by vasculitis (Figures 2(a) and 2(b)). It should be noted that she had an EGD three months prior to this event for refractory gastroesophageal reflux disease that was completely normal. In addition to her GI symptoms, patient also developed acute renal insufficiency with peak creatinine of 2.9 that eventually resolved before her discharge.

Based on recommendations from dermatology, patient was started on prednisone as well as dapsone for treatment of urticarial vasculitis. Her rash and her GI symptoms improved over the next few weeks, with complete resolution by week 4. A follow-up small bowel enteroscopy performed 2 months later showed a mild to moderate smooth stricture in D3 that was traversed with a pediatric colonoscope and no dilation was required. Repeat endoscopic biopsies showed lamina propria fibrosis, acute and chronic inflammation, and regenerative epithelial changes.

## 3. Discussion

Urticarial vasculitis is a rare vasculitis with skin urticaria, cutaneous small vessel involvement, and sometimes systemic manifestations [2]. In our patient, given the negative serologies for an autoimmune, infectious, and neoplastic process, the urticarial vasculitis is presumably due to the acute infection with her leg wound or associated with a drug reaction.

Our patient presented with GI symptoms as her initial extracutaneous manifestation of UV. However, urticarial vasculitis can affect various organ systems and patients can present with systemic symptoms including uveitis, episcleritis, angioedema, arthralgia, proteinuria, renal failure, and pulmonary disease [2, 6]. Mehregan et al. found that patients who have low complement level are more likely to have systemic symptoms [6].

Treatment of urticarial vasculitis is based on disease severity. Dapsone, colchicine, indomethacin, or hydroxychloroquine can be used for cutaneous disease in UV [2]. Corticosteroids are often necessary when patients present with extracutaneous manifestations of UV; azathioprine, cyclosporine A, cyclophosphamide, and mycophenolate mofetil can be used for those with steroid-refractory disease or severe systemic involvement [2]. In recent years, several authors have reported success in using Omalizumab, an anti-IgE monoclonal antibody, to treat patients who have refractory disease despite steroid and immunosuppressants [7–9].

## 4. Conclusion

We report a case of urticarial vasculitis-induced intestinal ischemia and emphasize the importance of early recognition of the various types of clinical manifestations of urticarial vasculitis. Prompt management of UV can prevent potentially life-threatening complications.
